# Dose-Dependent Von Willebrand Factor Inhibition by Aptamer BB-031 Correlates with Thrombolysis in a Microfluidic Model of Arterial Occlusion

**DOI:** 10.3390/ph15121450

**Published:** 2022-11-22

**Authors:** Susan M. Shea, Kimberly A. Thomas, Rassam M. G. Rassam, Emily P. Mihalko, Christina Daniel, Bruce A. Sullenger, Philip C. Spinella, Shahid M. Nimjee

**Affiliations:** 1 Division of Critical Care, Department of Pediatrics, Washington University in St. Louis School of Medicine, St. Louis, MO 63110, USA; 2 Trauma and Transfusion Medicine Research Center, Department of Surgery, University of Pittsburgh, Pittsburgh, PA 15213, USA; 3 Department of Surgery, Duke University Medical Center, Durham, NC 27710, USA; 4 Department of Neurological Surgery, The Ohio State University Medical Center, Columbus, OH 43210, USA

**Keywords:** microfluidics, ischemic stroke, aptamers, nucleotide, von Willebrand Factor, platelets

## Abstract

Von Willebrand Factor (VWF) plays a critical role in thrombus formation, stabilization, and propagation. Previous studies have demonstrated that targeted inhibition of VWF induces thrombolysis when administered in vivo in animal models of ischemic stroke. The study objective was to quantify dose-dependent inhibition of VWF-platelet function and its relationship with thrombolysis using BB-031, an aptamer that binds VWF and inhibits its function. VWF:Ac, VWF:RCo, T-TAS, and ristocetin-induced impedance aggregometry were used to assess BB-031-mediated inhibition of VWF. Reductions in original thrombus surface area and new deposition during administration of treatment were measured in a microfluidic model of arterial thrombolysis. Rotational thromboelastometry was used to assess changes in hemostasis. BB-031 induced maximal inhibition at the highest dose (3384 nM) in VWF:Ac, and demonstrated dose-dependent responses in all other assays. BB-031, but not vehicle, induced recanalization in the microfluidic model. Maximal lytic efficacy in the microfluidic model was seen at 1692 nM and not 3384 nM BB-031 when assessed by surface area. Minor changes in ROTEM parameters were seen at 3384 nM BB-031. Targeted VWF inhibition by BB-031 results in clinically measurable impairment of VWF function, and specifically VWF-GPIb function as measured by VWF:Ac. BB-031 also induced thrombolysis as measured in a microfluidic model of occlusion and reperfusion. Moderate correlation between inhibition and lysis was observed. Additional studies are required to further examine off-target effects of BB-031 at high doses, however, these are expected to be above the range of clinical targeted dosing.

## 1. Introduction

The search persists for a safe and efficacious treatment for arterial thrombosis in the event of acute myocardial infarction or ischemic stroke. In the United States (US), approximately 795,000 people per year experience a stroke, with the vast majority (87%) being ischemic. Stroke is the most common cause of severe long-term disability. The total cost of stroke in the US is estimated to be over $50 billion annually [1]. 

The only drug currently approved to treat acute ischemic stroke (AIS) caused by pathological thrombus formation is recombinant tissue plasminogen activator (rtPA), which targets lysis of fibrin-rich clots via conversion of plasminogen into its active form, plasmin. Plasmin cleaves fibrin, thereby breaking down occlusive clot structure. rtPA has several limitations that impede its safety and efficacy in treating patients who present with AIS: (i) it has a narrow temporal window of use that excludes >90% of ischemic stroke patients [2], (ii) treatment results in symptomatic intracranial hemorrhage (sICH) in ~6% of patients, resulting in significant morbidity and mortality, (iii) it achieves recanalization in only around 10% of patients who present with large vessel occlusion (LVO) stroke (15% of AIS patients) [3] and 30% of all AIS patients, and (iv) re-occlusion often occurs after treatment [4]. Endovascular thrombectomy (EVT) is a minimally invasive means of removing a thrombus blocking the intracranial internal carotid artery, middle cerebral artery (MCA) or anterior cerebral artery (ACA), but can only be performed in patients who present with LVO stroke. LVO stroke disproportionately results in poor outcomes compared to other stroke etiologies, and up to 90% of stroke-induced mortality occurs in patients with LVO stroke [5]. Intravenous thrombolysis therapy (IVT) with tPA can be used in isolation or as a bridge to EVT [6].

The mechanism of thrombosis responsible for thromboembolic stroke comprises elongation of von Willebrand Factor (VWF) on an extravascular surface (i.e., subendothelial collagen), resulting in pathological capture of platelets at high shear [7]. Progressive vessel occlusion causes stagnation and potentiates coagulation. While cardioembolic strokes are canonically fibrinous due to formation in zones of stagnation in the left atrium, they may develop a VWF-platelet-rich outer shell once situated in the arterial setting post-embolization [8]. The thrombi responsible for vessel occlusion in stroke of both thromboembolic and cardioembolic stroke may therefore be resistant to rtPA-mediated lysis. Attacking these targets on outer the shell of the thrombus may facilitate recanalization of the vessel.

BB-031 is a ribonucleic acid (RNA) aptamer that binds to the VWF A1 domain (note: in previous publications, BB-031 is called DTRI-031, and these two names refer to the same aptamer) [9]. Aptamers are single-stranded oligonucleotides that have high target binding affinity and can be rapidly reversed by their antisense counterpart, making them well suited for anticoagulant and antithrombotic applications [10]. Pre-clinical development of BB-031 has demonstrated it to be a novel, rapid-onset, and rapidly reversible antithrombotic agent in both in vitro assays and in vivo animal models [9]. Interestingly, BB-031 also has thrombolytic properties, and resulted in recanalization post-occlusion in a canine model of stroke [9]. As BB-031 targets VWF A1 domain-platelet Glycoprotein Ib (GPIb) interactions, and not systemic induction of fibrinolysis as does rtPA, this limits the mechanism of treatment to that of arterial thrombosis and therefore would be a highly desirable alternative to current therapeutic approaches. Furthermore, rapid reversibility allows the treatment to be arrested, which is impossible with rtPA. BB-031 was tested in a recently completed Phase I clinical trial (NCT05005520) to assess safety in healthy volunteers.

The objectives of this study were to determine (i) optimal in vitro BB-031 dosing in a microfluidic model of thrombolytic efficacy, (ii) how thrombolytic efficacy correlates with measures of inhibition in clinical assays that are readily available for therapeutic direction, and (iii) which currently commercially available assay best capitulates dose-dependent functional effects of BB-031.

## 2. Results

### 2.1. Dose-Dependent Inhibition of VWF by BB-031 Can Be Measured by Clinically and Commercially Available Assays

**Figure 1:**
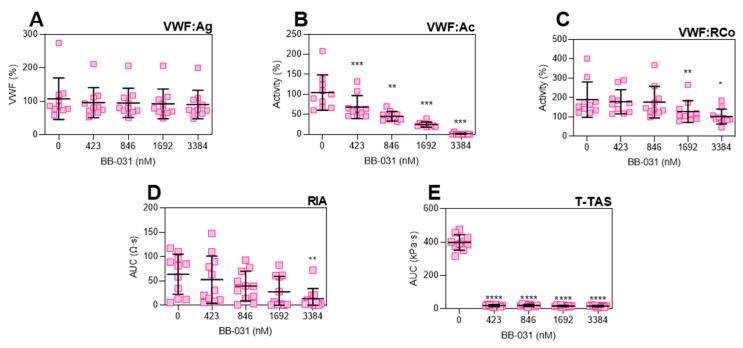
VWF function panel. Dose–response of (**A**) VWF:Ag, (**B**) VWF:Ac, (**C**) VWF:RCo, (**D**) Ristocetin-induced whole blood impedance aggregometry (RIA), and (**E**) T-TAS. *N* = 10. Individual data points are shown. Error bars represent mean ± standard deviation (SD). **** *p* < 0.0001; *** *p* < 0.001; ** *p* < 0.01, * *p* < 0.05.

**Figure 2:**
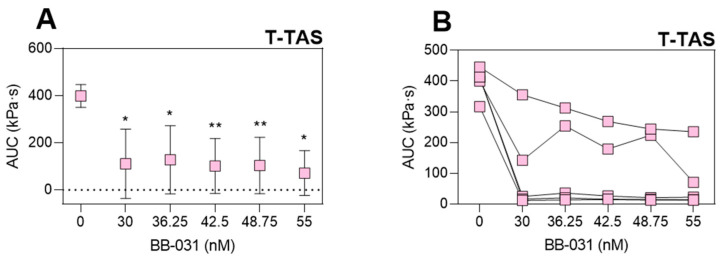
T-TAS inhibition at lower concentrations of BB-031. As function was maximally inhibited at the lowest dose of drug in the selected dose response curve, another dose–response curve for this assay alone was generated. The same data are shown as (**A**) summary data (mean ± standard deviation (SD) and (**B**) individual data points. *N* = 5. ** *p* < 0.01, * *p* < 0.05.

VWF antigen levels are shown in Figure 1A, and as expected were unchanged by addition of BB-031. All VWF functional assays were inhibited by BB-031 (Figure 1B–E). VWF:Ac was maximally inhibited at the highest dose (9/10 of values were below the detectable limit; Figure 1B). VWF ristocetin cofactor assay (VWF:RCo) demonstrated inhibition at only high doses of drug, and did not exhibit a complete loss of function at the highest dose (Figure 1C). Ristocetin impedance aggregometry (RIA) uses the antibiotic ristocetin to help VWF binding to the GPIb/IX/V complex and is therefore dependent on both functional VWF and platelets [11]. In the presence of escalating doses of BB-031, RIA demonstrated decreases in area under the curve (AUC) in response to dose but was only statistically significantly different from vehicle at the highest dose (Figure 1D). T-TAS AUC did not increase above the initial pressure reading in the assay at all doses of BB-031 (Figure 1E). An additional dose–response was performed on the T-TAS device with even smaller doses of drug on a subset of donors (*N* = 5; Figure 2), and 3/5 donors lost function on this assay at a 30 nM BB-031 dose. Complete blood counts (CBCs) were performed on all donors and were within normal ranges (Table S1).

### 2.2. Off-Target Coagulopathy Induction by BB-031 Was Not Detected by Viscoelastic Testing

**Figure 3:**
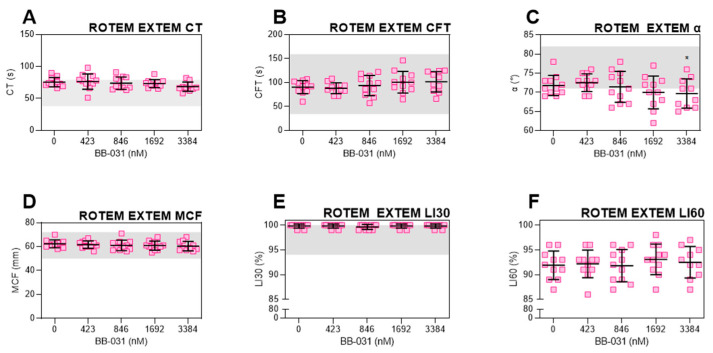
Bleeding risk assessment. ROTEM EXTEM was performed to assess the risk of bleeding with increasing doses of BB-031. Selected ROTEM parameters are displayed: (**A**) clotting time (CT), the start of measurement until amplitude reaches 2 mm, (**B**) clot formation time (CFT), the time between initiation of clotting until clot firmness reaches 20 mm amplitude, signifying fibrin polymerization and clot stabilization, (**C**) alpha angle (α), the angle between the x axis and tangential line to the curve at 2 mm, describing clot kinetics, (**D**) maximum clot firmness (MCF), the highest amplitude reached reflecting maximal clot strength, (**E**) lysis index at 30 min (LI30), and (**F**) lysis index at 60 min (LI60s). Both LI30 and LI60 describe the progress, where 100% reflects no lysis progression. *N* = 10. Individual data points are shown. Error bars represent mean ± standard deviation (SD). Gray bands represent reference ranges for CT, CFT, MCF, α, and LI30 in healthy adults (instructions for use (IFU) and PMID 15870552). Reference range is not available for LI60. * *p* < 0.05.

ROTEM was used to assess any defects in hemostasis, which could indicate a potential for clinical bleeding risk (Figure 3). Clotting time (CT) decreased slightly (Figure 3A), representing a faster time to initial fibrin formation, while clot formation time (CFT) conversely increased by approximately 10 s, potentially representing a slower fibrin crosslinking, though the increase in CFT was not statistically significant and remained in the normal range (Figure 3B). BB-031 at the highest dose had α angle values significantly decreased from vehicle, indicating potential inhibition of fibrin function or cross-linking (Figure 3C). Maximum clot firmness (MCF), lysis index at 30 min (LI30), and lysis index at 60 min (LI60) parameters were also not impacted by dose indicating no differences in clot strength and, importantly, fibrinolysis (Figure 3D–F). 

### 2.3. BB-031 Induces Recanalization in a Microfluidic Model of Occlusion and Post-Occlusion Attempted Perfusion

**Figure 4:**
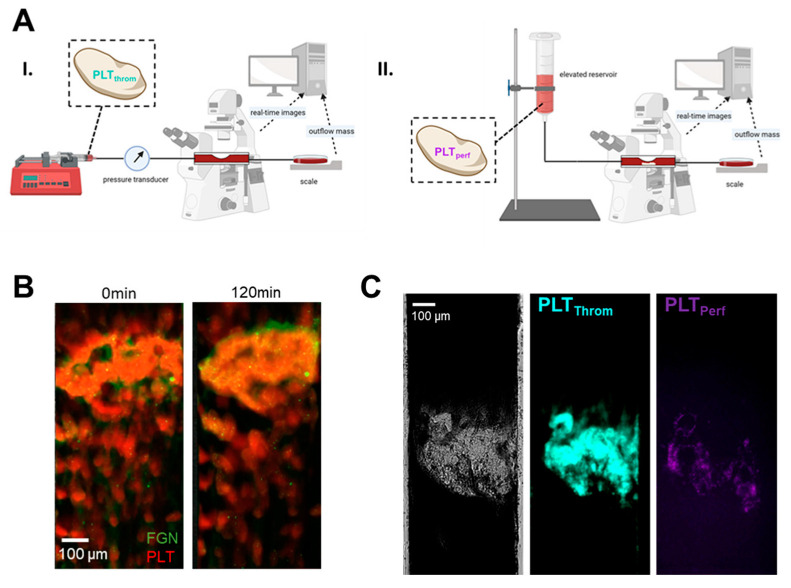
Microfluidic reperfusion experiments. (**A**): I. Partial occlusion is induced in a controlled flow setting in a microfluidic stenosis (initial wall shear rate at the throat 3500 s^−1^; target pressure increase 25 mmHg). Platelets in this sample are labelled aqua (PLT_throm_). II. Reperfusion is initiated with a treated sample using the syringe pump and low flow. Once the treated sample reaches the testbed a constant arterial pressure head is induced using gravity to simulate in vivo flow conditions (i.e., true occlusion). Platelets in the reperfusion sample are labelled purple (PLT_perf_). Created with BioRender.com, accessed on 21 October 2022. (**B**): Control thrombus with platelets stained red and fibrin(ogen) (FGN) stained green, immediately post occlusion (0 min, left) and after 120 min (right). (**C**): Representative images during perfusion in II. At left, grayscale transmitted light. Center, aqua PLT_throm_. Right, new purple deposition of PLT_perf_.

**Figure 5:**
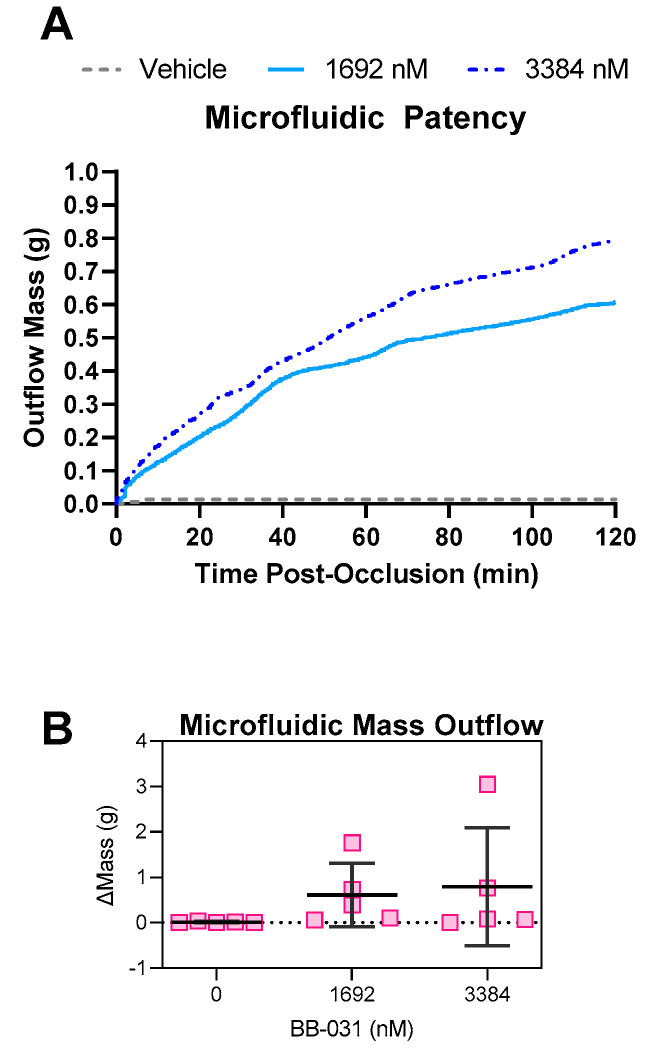
Microfluidic Patency. Outflow of the microfluidic channel was measured using a scale to determine the patency of the channel and assess recanalization. No change in mass represents no flow (not patent/occluded). (**A**) Mean traces are shown for vehicle, treatment with 1692 nM BB-031, and treatment with 3384 nM BB-031, *N* = 5 each. The raw mass output was smoothed using a LOESS function to account for erroneous jumps due to movement or instability external to the experiment. (**B**) The change in mass was calculated by subtracting the last mass value from the first mass value Individual data points are shown in (**B**). Error bars represent mean ± standard deviation (SD).

The microfluidic setup is demonstrated in Figure 4. Partial occlusion is induced in a microfluidic collagen-coated stenosis (3500 s^−1^), which is then reperfused via constant pressure with treated blood from the same donor. Platelets in the original thrombus were labelled with one fluorophore, and platelets in the dosed post-occlusion perfusion sample with another to allow quantification of both lysis and new deposition. Outflow of the chamber to determine patency (i.e., open and unobstructed by occlusive thrombus) of the stenotic channel was determined by measuring the outflow mass.. The mean mass outflow traces are shown in Figure 5A, and total mass increase in Figure 5B. Patency was achieved in 60% (3/5) of samples reperfused with 1692 nM BB-031, 40% (2/5) of samples with 3384 nM BB-031, and 0% (0/5) of samples perfused with vehicle. Notably, when treated with BB-031, patency was achieved and maintained within the first minute of reperfusion due to lack of immediate deposition of new thrombus, as occurs in the vehicle-treated case. These differences, while trending, were not statistically significant.

**Figure 6:**
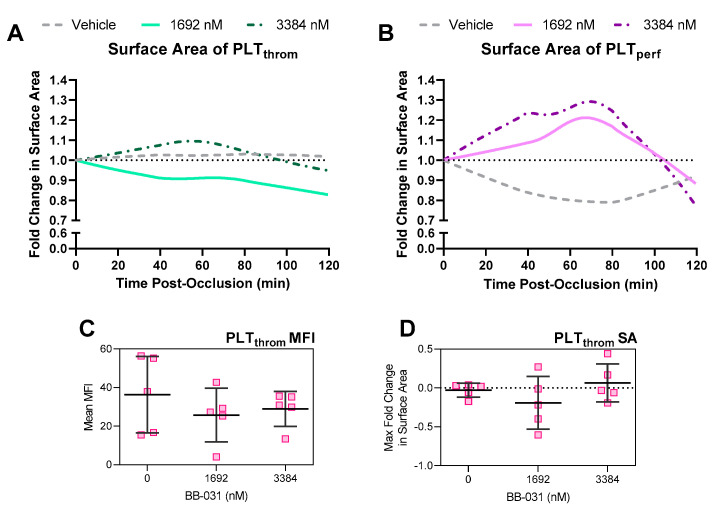
Platelets involved in initial thrombus formation were labeled with a fluorophore excited by an ultraviolet (UV) wavelength (350 nm, PLT_throm_), platelets in the treated reperfusion sample were labelled with a fluorophore excited by a near far red wavelength (646 nm, PLT_perf_). Surface area was determined using fluorescence microscopy images in post processing using a thresholding function. Reperfusion samples were treated with either vehicle, 1692 nM BB-031, or 3384 nM BB-031. Data are shown as traces representing mean Loess curves of *N* = 5 experiments. (**A**) Fold change in surface area of PLT_throm_ during reperfusion. (**B**) Fold change in surface area of PLT_perf_ during reperfusion. Quantitative microfluidic parameters describing thrombolytic efficacy were also derived. (**C**) Mean PLT_throm_ mean fluorescence intensity (MFI), used in correlation analysis as a measure of microfluidic lytic efficacy (MLE). *N* = 5. (**D**) Maximum fold change in PLT_throm_ surface area (SA). Individual data points are shown in (**C**,**D**). Error bars represent mean ± standard deviation (SD).

Fold change in surface area (SA) of the original thrombus (thrombus platelets, “PLT_throm_”) from the start of treatment (vehicle or BB-031) perfusion post-occlusion is shown in Figure 6A. Fold change in SA of post-occlusion platelets (perfusion platelets, “PLT_perf_”; i.e., deposition of new thrombus after occlusion) are shown in Figure 6B. PLT_throm_ SA changed by +1.8% (1.41%) (mean (standard error of the mean (SEM)) over 120 min when perfused with vehicle, −17.20% (13.93%) when perfused with 1692 nM BB-031 (*p* = 0.096 vs. vehicle), and −5.17% (7.41%) when perfused with 3384 nM BB-031 (*p* = 0.482 vs. vehicle, *p* = 0.0134 vs. 1692 nM). Interestingly and unexpectedly, while BB-031 treatment allowed flow, this also came with deposition of new platelets, and in greater magnitude at the highest dose. SA of PLT_perf_ maximally decreased around 80 min post occlusion by 10.76% (6.68%) when perfused with vehicle, but maximally increased by 30.23% (21.83%) when perfused with 1692 nM BB-031 (*p* = 0.028 vs. vehicle), and 65.81% (36.73%) when perfused 3384 nM BB-031 (*p* = 0.067 vs. vehicle, *p* = 0.002 vs. 1692 nM). However, patency was maintained as shown in Figure 5, and PLT_perf_ completely lysed away by 120 min with BB-031 administration at both doses. 

Quantitative parameters were derived from microfluidic reperfusion experiments (Figure 6C,D). Average PLT_throm_ mean fluorescence intensity (MFI) extracted from the entire curve is shown in Figure 6C. Maximum fold change in PLT_throm_ SA is shown in Figure 6D. Treatment with 1692 nM BB-031 resulted in a greater reduction in SA than both vehicle and 3384 nM BB-031, and treatment with either dose had an approximately equivalent reduction in mean PLT_throm_ MFI. However, no comparisons were statistically significantly different. Average PLT_throm_ MFI had less variation than max fold change in SA and was therefore used to represent microfluidic lytic efficacy in correlation analyses.

### 2.4. VWF Inhibition Correlates with Thrombolysis

**Figure 7:**
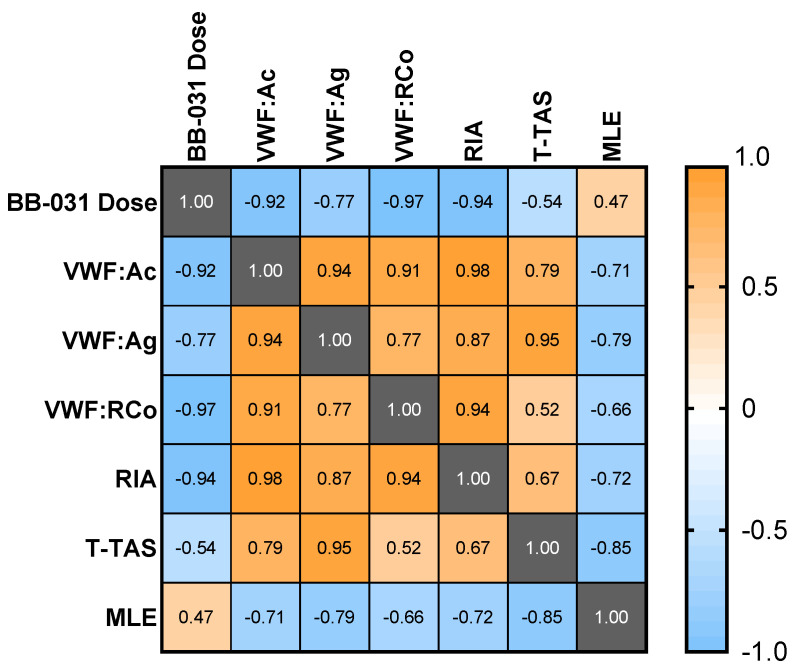
Correlation matrix of assay parameters. Pearson correlation coefficients (ρ) calculated among paired samples and visualized in a correlation matrix. RIA: Ristocetin Impedance Aggregometry; MLE: microfluidic lytic efficacy.

Dose, measures of functional inhibition (Figure 2), and microfluidic lytic efficacy (MLE; derived from Figure 6C) were correlated and visualized in Figure 7. All assays except MLE correlated negatively with dose. Measures of functional inhibition all correlated positively amongst themselves, and all correlated negatively with MLE, indicating enhanced thrombolysis is concomitant with increased inhibitory effect of BB-031 on VWF function. VWF:Ac, VWF:RCo, and ristocetin-induced whole blood impedance aggregometry all had correlation coefficients |ρ| > 0.9 with dose. T-TAS correlated weakest with dose (ρ = −0.54). MF thrombolytic efficacy correlated best with T-TAS (ρ = −0.85), VWF:Ag (ρ = −0.89), and VWF:Ac (ρ = −0.79), and coefficients were slightly lower for VWF:Ac (ρ = −0.71), ristocetin-induced whole blood impedance aggregometry (ρ = −0.72), and VWF:RCo (ρ = −0.66).

## 3. Discussion

Treatment with BB-031 in healthy human whole blood samples in vitro resulted in inhibition of VWF function detectable by various clinical assays: Siemens VWF:Ac, DiaPharma T-TAS, Chrono-Log VWF:RCo, and Chrono-Log RIA (Figure 1). Specifically, BB-031 inhibition of platelet GPIbα binding is dose-dependent and measurable on clinically available assays. Dosing with BB-031 did not alter VWF antigen levels, as expected (Figure 1A). Furthermore, inhibitory effects were well preserved during sample handling and processing, including sample storage at −80 °C, shipment, and thawing. Additionally, of note, function was preserved during drug preparation using 0.45% saline alone at room temperature (no heat nor buffers were applied, as they had been for pre-clinical formulations of BB-031). Interestingly, sensitivity varied across assays, with the T-TAS assay being remarkably sensitive to inhibition by BB-031 at very low doses. As this is the only assay in our clinical panel that incorporates flow, we hypothesize that this sensitivity is due to the both the shear-induced exposure of additional A1 domains that are then inhibited by available aptamer, as well as the mechanistically necessary release of additional VWF from platelet alpha granules that participate in thrombosis [12], which would also be inhibited by available aptamer.

BB-031 did not induce fibrinolysis, as expected (Figure 3E,F). This is supportive of its advantageous use over rtPA. Interestingly, only α angle was statistically significantly different from baseline at the highest dose (Figure 3D), demonstrating a very slight decrease in clot formation kinetics as measured by viscoelastic testing. CT trended down, which may indicate faster thrombin generation and initial fibrin formation, yet CFT trended upwards. Both remain within the healthy reference range and are not clinically-relevant changes. The mechanism of these slight effects may be due to off-target inhibition of either platelets mediated by VWF A1, or inhibition of other VWF binding sites, such as the D’-D3 region, which binds FVIII. However, as ROTEM parameters have yet to be fully translated to rtPA-treated patients, we cannot make outcome predictions.

BB-031 induced thrombolysis in a microfluidic model of reperfusion of a nearly occlusive arterial thrombus, as shown in both trends in patency (Figure 5) and in reductions in thrombus surface area (Figure 6) relative to treatment with vehicle. The microfluidic reperfusion system has high (patho)physiological fidelity, as thrombosis is induced in an arterial setting on a collagen-coated stenosis, simulating the post-plaque rupture environment and inducing the resulting catastrophic acute event of vessel occlusion. To our knowledge, this system is the closest in vitro microfluidic model approximating thrombotic occlusion and treatment. Loyau et al. took a similar approach to study rtPA-induced fibrinolysis under flow, but focused on the induction of coagulation to form fibrin-rich clots, perfused them at venous and a lower arterial shear rate (1500 s^−1^) for only 10 min [13]. Wang et al. also studied urokinase-type plasminogen activator (uPA) thrombolysis in vitro in an endothelialized system, but again focused on non-occlusive fibrin-rich clots and utilized shorter timescales (~35 min) than our study [14]. The thrombi in our system are mechanistically VWF-dependent and platelet-rich (Figure 4) as they are formed at an initial wall shear rate of 3500 s^−1^, and our post-occlusion reperfusion time was much longer (120 min), selected based on timescales seen in animal models where BB-031 induced recanalization [9]. Kim and Shea et al. previously published a macro-scale glass capillary tube to induce high shear thrombosis, and reperfused thrombi with potential lytic agents [15]. In this system, we advance our approach via (i) perfusing post-occlusion with autologous dosed blood instead of saline, (ii) a microfluidic model to reduce volume needs allowing higher throughput, (iii) quantification of thrombi using fluorescent microscopy, and (iv) reperfusion with a gravity pressure head instead of a constant flowrate. Reperfusion post-occlusion with constant pressure better recapitulates systemic administration of drug that is delivered in vivo to a site of ischemia. While BB-031 has previously been shown to inhibit thrombosis in vitro in canine whole blood using the T-TAS, we for the first time demonstrate inhibition in human samples in various assays, demonstrate that inhibition of BB-031 survives a freeze–thaw cycle in plasma, and demonstrate microfluidic thrombolysis, both informing on clinical use of BB-031 and recapitulating recanalization observed by Nimjee et al. in vivo in canines [9]. Other VWF A1 domain-targeting aptamers have been developed, such as ARC 1779 [16] and TAGX-004 [17], and have been evaluated both in thrombo-prevention and thrombocytopenia purpura (TTP). In this study, we tested the hypothesis that BB-031 is a VWF-platelet-specific thrombolytic agent, furthering the study of the previous in vivo recanalization findings [9]. Of note, ARC 1779 and TAGX-004 have not been evaluated as recanalization agents. 

In our microfluidic system, surprisingly, treatment with 1692 nM BB-031 was more efficacious than treatment with 3384 nM BB-031. Treatment with the lower dose resulted in greater lysis of the original thrombus (reduction in PLT_throm_ SA), as well as better inhibition of deposition of new thrombus (reduced PLT_perf_ fold change; Figure 6). Interestingly, inhibition correlated with dose as expected in non-flow based assays (e.g., VWF:Ac, which was maximally inhibited at the highest dose). Treatment with vehicle resulted in a stable, reinforced thrombus that did not change throughout reperfusion, as represented by complete stagnation throughout the reperfusion period (no patency; Figure 5) and the accompanying expected lack of surface area reduction and lack of significant deposition of new thrombus due to lack of flow (Figure 6). The surface area of new deposition decreased from baseline in the setting of reperfusion with vehicle, which is attributable to retraction in the setting of stagnation. While surface area reduction was not extreme, clear differences in patency were observed. Reductions in the height of the thrombus were not captured, a limitation of 2D image acquisition, and are likely occurring and responsible for the improvements in flow. Off-target effects (e.g., inflammatory pathways [18]) may be responsible for the surprising reduction in thrombolytic and inhibitory efficacy observed at the highest dose, as well as the effects seen on ROTEM. However, the highest dose selected in this study was chosen intentionally to be at the maximal potential clinical dose. Further exploration of the mechanism of these effects may help inform on clinical safety. 

While dose best correlated with VWF:RCo (ρ = −0.97) and ristocetin-induced whole blood impedance aggregometry (ρ = −0.97), VWF:Ac had a high correlation coefficient (ρ = −0.92), a log-linear dose response, and was maximally inhibited at the highest dose. It may therefore be an ideal clinical measure of inhibition, however this assay has limitations: (i) it requires processing of the sample into platelet-poor plasma (PPP), which may increase turnaround times relative to a whole blood assay, and (ii) it is neither FDA-approved nor yet available in the US at the time of this writing. Incredibly low doses of drug (≤30 nM, two orders of magnitude less than an equivalent 0.25 mg/kg in vitro dose) completely inhibited the T-TAS assay. While this potentially indicates complete inhibition of VWF-platelet-mediated thrombosis under flow, other assays such as VWF:Ac may better provide information regarding the level of molecular VWF inhibition, especially if and when BB-031 is administered clinically as a thrombolytic agent. However, T-TAS could still be of potential clinical utility for BB-031 monitoring as it most directly represents thrombotic function of the assays used in this study. MLE correlated weakly with dose, due to the highest dose being less efficacious than the lower dose in the model of reperfusion in vitro. However, MLE still correlated well with the other assays of inhibition. The negative correlation coefficients indicate that enhanced thrombolysis corresponds to greater inhibition of VWF, suggesting that the thrombolytic efficacy of the drug may also be captured clinically in an assay of VWF functional inhibition. 

There are a handful of limitations to this study. First, as these are healthy volunteer samples dosed with drug ex vivo, they likely do not fully encompass the effects of in vivo intravenous administration, nor the blood state or local milieu in a patient experiencing a stroke. Furthermore, while cardioembolic strokes may have VWF-platelet rich structure in addition to a fibrin-rich core presumed to be due to the origin of the thrombus [8], this system is more directly applicable to thromboembolic stroke. Chamber rigidity, perfusion immediately post thrombus formation, along with other in vitro factors may also hinder the physiological relevance of the assay. As the primary goal of this study was to determine dose–response of BB-031 and assess its efficacy in the microfluidic model, we did not compare head-to-head with current clinically used formulations of rtPA. Given the promising findings in this study, studies comparing lytic efficacy of alteplase and tenecteplase with BB-031 and other design modifications to address the above limitations would be worthwhile future endeavors. 

## 4. Materials and Methods

### 4.1. Sample Collection

Whole blood was collected from healthy volunteer donors (*N* = 10) in anticoagulated collection tubes (sodium citrate or Benzylsulfonyl-D-Arg-Pro-4-amidinobenzylamide (BAPA; a synthetic inhibitor of FXa and thrombin), depending on assay instructions for use (IFU)), or green-top heparin vacutainers for use in microfluidic models (Washington University in St. Louis (WUSTL) Institutional Review Board (IRB) Approved, Study #202105078). Donors returned for a second collection to allow for paired analyses; all assays were run using samples from the same donor pool. Immediately post collection, a CBC was performed using a hematology analyzer (Advia 2120i; Siemens, Munich, Germany). Samples were either used immediately for whole blood functional assays, or further processed into PPP and stored at −80 °C for batched analyses, per assay IFU. Prior to assay or the above mentioned further processing, samples were aliquoted and dosed with 423 nM, 846 nM, 1692 nM, or 3384 nM of BB-031, which correspond to 0.25 mg/kg, 0.5 mg/kg, 1 mg/kg, and 2 mg/kg, respectively, in vivo. Vehicle controls (addition of same volume of normal saline) were also run. Dosed samples were allowed to incubate for 5 min before further handling.

### 4.2. VWF:Ag

Frozen citrated dosed PPP samples (*N* = 10) were thawed in a 37 °C water bath for 5 min, and then run on a CompactMax (Diagnostica Stago, Parisppany, NJ, USA) using STA^®^-Liatest^®^ VWF:Ag, an immuno-turbidimetric assay for measuring VWF antigen levels. Antigen levels are reported as a percentage (%) relative to a control level as determined by the test-provided controls and CompactMax software.

### 4.3. VWF:Ac

Frozen citrated dosed PPP samples (*N* = 10) were shipped on dry ice to Dr. David Lillicrap’s lab at Queen’s University, Kingston, Ontario, Canada. The Innovance^®^ “VWF:Ac” assay (Siemens) measures GPIb binding via the addition of recombinant GPIb and anti-GPIb polystyrene particles. VWF binding to particles results in agglutination which is measured turbidimetrically. The assay was run using a BCSXP 1500 (Siemens). Data are reported as a percentage (%) of activity.

### 4.4. T-TAS

The T-TAS is a commercialized microfluidic device comprising an automated microfluidic constant-flow rate driven system [19]. Whole blood samples (*N* = 10) were collected into 3 mL BAPA tubes, then dosed with BB-031. Dosed samples were run on PL-Chips (DiaPharma, West Chester Township, OH, USA) per manufacturer’s instructions. In brief, samples were loaded into reservoirs which were then capped, inverted, attached to the chip, and perfused using the accompanying software for up to 10 min (assay time limit). Data are reported as AUC of the pressure in the channel over time.

### 4.5. VWF:RCo

VWF-Ristocetin cofactor assay (VWF:RCo) was performed using a kit containing lyophilized platelets and reagents (Chrono-Log, Havertown, PA, USA) via light transmission aggregometry (LTA). Frozen citrated dosed PPP samples (*N* = 10) were thawed as above and run per manufacturer’s instructions. In brief, VWF:RCo activity is determined by agglutination of standard suspensions of lyophilized human platelets with sample plasma in the presence of ristocetin. Both normal and deficient reference human plasma are provided for generation of a standard curve and quality control, respectively. Data are reported as a percentage (%) of activity as determined relative to the standard curve, calculated by the Chrono-Log software.

### 4.6. Ristocetin Induced Whole Blood Aggregometry (RIA)

Citrated dosed whole blood (*N* = 10) was used for ristocetin-induced whole blood impedance aggregometry. The assay was run per manufacturer’s instructions. In brief, dosed whole blood samples were mixed with pre-warmed sodium chloride. Ristocetin (Chrono-Log) was added such that the final concentration was 1 mg/mL, and aggregation recorded using the accompanying software. Data are reported as AUC of the aggregation trace.

### 4.7. ROTEM

Citrated dosed whole blood (*N* = 10 was used for ROTEM (Werfen Worldwide, Bedford, MA, USA). The assay was performed using the EXTEM (extrinsic pathway) agonist per manufacturer’s instructions. Data reported from this assay include clotting time (CT, s), clot formation time (CFT, s), maximum clot formation (MCF, mm), alpha angle (α, °), lysis index at 30 min (LI30, %), and lysis index at 60 min (LI60, %).

### 4.8. Microfluidics

A micromachined mold comprising 8 flow chambers with stenotic regions of interest was used to fabricate polydimethyl siloxane (PDMS) negatives, which were bonded to glass slides using a plasma cleaner (Harrick Plasma, Ithaca, NY, USA). Individual flow chambers were 480 µm wide, and the height at the stenosis was 80 µm. Chambers were incubated with 9:1 0.9% sodium chloride:collagen (equine fibrillar type I, Chrono-Log) overnight and rinsed with phosphate-buffered saline (PBS) prior to perfusion. Whole blood (*N* = 5) not treated with BB-031 nor vehicle was incubated with a monoclonal CD41 antibody conjugated to DyLight 350 (Novus Biologicals, Littleton, CO, USA; catalog no. NB100-2614UV; labeling original thrombus platelets, “PLT_throm_”) and perfused through the stenotic microfluidic device to induce pathophysiologically-relevant arterial thrombosis (initial wall shear rate 3500 s^−1^ (flowrate 0.108 mL/min); system previously described in [20]). A separate aliquot of whole blood from the same donor was incubated with the same clone of CD41 conjugated to Janelia Fluor 646 (Novus Biologicals; catalog no. NB100-2614JF646; labeling new perfusion platelets, “PLT_perf_”) and dosed with either vehicle (normal saline), 1692 nM BB-031, or 3384 nM BB-031. The second aliquot was delivered upstream without disturbing the original thrombus, gently perfused to the site of thrombus using a syringe pump, and then switched to constant-pressure perfusion using gravity and an arterial pressure head which would induce a shear rate of 3500 s^−1^ in an unobstructed identical channel. A schematic of the approach is shown in Figure 4. AF488 fibrinogen (Fisher Scientific, Pittsburgh, PA, USA; 0.01 mg/mL) was also added to a healthy control along with PLT_throm_ labelling as described above, perfused until occlusion, and imaged after two hours of retraction to visualize platelet and fibrinogen content in the thrombus (Figure 4B). Outflow of blood through the flow chamber was measured using a downstream scale. Upon occlusion, there is no flow out of the chamber, and the mass slows to a stop, first remaining constant but then shortly resulting in evaporation of the outflow and a concomitant decrease in mass over the remaining duration of the experiment. A constant mass was assumed if mass was decreasing to approximate a correction for evaporation. Total change in corrected mass was reported as the end value (maximal total outflow).

Experiments were imaged in real time (framerate 2/s) using fluorescence microscopy. Images of reperfusion were thresholded using Python (version 3.10.0) to yield surface area over time of the original thrombus (PLT_throm_) and any new deposition (PLT_perf_), and Loess curves were used to smooth variation in fluorescence data due to artifacts. Fold change was calculated by dividing surface area at each timepoint by the surface area at the first timepoint of drug or vehicle dosed WB perfusion, i.e., after thrombus had formed. MFI was also derived from unaltered (i.e., no changes in histogram curves applied) original data files and fold change was calculated in the same fashion. The average MFI across all biological replicates was derived to yield MLE values for use in correlation analyses.

### 4.9. Analysis

Samples from the same donor were assumed paired. Data within each assay were analyzed using a One-way Analysis of Variance (ANOVA) using the Geisser-Greenhouse correction, which corrects for violations of the assumption of sphericity (inherent to ANOVA, assumes variance among treatments is equal). If the ANOVA was statistically significant (*p* < 0.05), the analysis proceeded with multiple comparisons (Dunnett’s test) of each dose against the untreated measurement. Data are visualized as mean, with error bars representing SEM unless otherwise described. Correlations were also performed among assays and among individual doses using Pearson’s correlation coefficient (ρ) and visualized using a correlation matrix.

## 5. Conclusions

BB-031, an aptamer that binds the VWF A1 domain resulting in inhibition of platelet binding via GPIb, was tested in vitro to examine dose response in clinically available assays of VWF functional inhibition as well as thrombolytic efficacy using a novel microfluidic approach to model thrombotic occlusion and reperfusion. Treatment with BB-031 resulted in inhibition captured on all VWF functional assessment platforms and did not induce a hyperfibrinolytic phenotype as measured by ROTEM. BB-031 treatment was also able to induce thrombolysis under flow, and unlike treatment with vehicle, achieved patency during reperfusion. Treatment with BB-031 induced slight effects on coagulation at the highest dose yet maintained values within the normal range, and the lack of hyperfibrinolysis and the reversibility of the drug highly support advantages of its use over rtPA. The highest dose tested (3384 nM, approximating 2 mg/kg) was less efficacious in the model of microfluidic reperfusion than the lower dose tested (1692 nM, approximating 1 mg/kg). Further investigation is needed to elucidate both coagulation inhibitory mechanisms and potential off-target effects at high concentrations of drug to fully optimize dosing, and subsequent in vivo testing is also needed to understand the potential consequences of these surprising effects. 

## Data Availability

Data supporting results can be obtained via written request via email to the corresponding author.

## Supplementary Materials

The following supporting information can be downloaded at: https://www.mdpi.com/article/10.3390/ph15121450/s1, Table S1: Donor CBCs. Complete blood counts (CBCs) expressed as mean (standard deviation (SD)) for all donors at their first and second donations.
