# Supplementary material for: Dose-Dependent Von Willebrand Factor Inhibition by Aptamer BB-031 Correlates with Thrombolysis in a Microfluidic Model of Arterial Occlusion
Source: Pharmaceuticals (Basel). 2022 Nov 22;15(12):1450. doi: 10.3390/ph15121450 (PMC9785393; doi:10.3390/ph15121450)
Supplement: Supplementary file 1 [file pharmaceuticals-15-01450-s001.zip › pharmaceuticals-1942088-supplementary.pdf]

Table S1. Donor CBCs. Complete blood counts (CBCs) expressed as mean (standard deviation (SD)) for all donors at their first and second donations.

**Parameter**	**Donation 1 (mean (SD))**	**Donation 2 (mean (SD))**
Platelet Count (*10^3 cells/μL)	174 (32)	189 (26)
Mean Platelet Volume (fL)	10.7 (0.6)	11.6 (0.3)
Hematocrit (%)	37.3 (1.7)	38.9 (1.2)
Hemoglobin (g/dL)	12.9 (0.7)	12.9 (0.5)
